# Reproducibility of slice-interleaved T_1_ (STONE) mapping sequence

**DOI:** 10.1186/1532-429X-18-S1-P51

**Published:** 2016-01-27

**Authors:** Steven Bellm, Tamer Basha, Long Ngo, Sophie Berg, Kraig V Kissinger, Beth Goddu, Warren J Manning, Reza Nezafat

**Affiliations:** 1 Radiology, Beth Israel Deaconess Medical Center, Boston, MA USA; 2 Medicine, Beth Israel Deaconess Medical Center, Harvard Medical School, Boston, MA USA

## Background

Slice interleaved T_1_ mapping sequence (*STONE*) allows quantification of native T_1_ of the entire ventricle in a single free-breathing scan. Using this sequence, data acquisition for different slices are interleaved, allowing longer recovery time after the inversion pulse, which could result in improved accuracy and precision. However, measurement reproducibility of the STONE sequence has not been previously studied. In this study, we sought to assess native T_1_ measurement reproducibility a) *within session*, b) *between sessions* and c) *between days*.

## Methods

**Figure 1:**
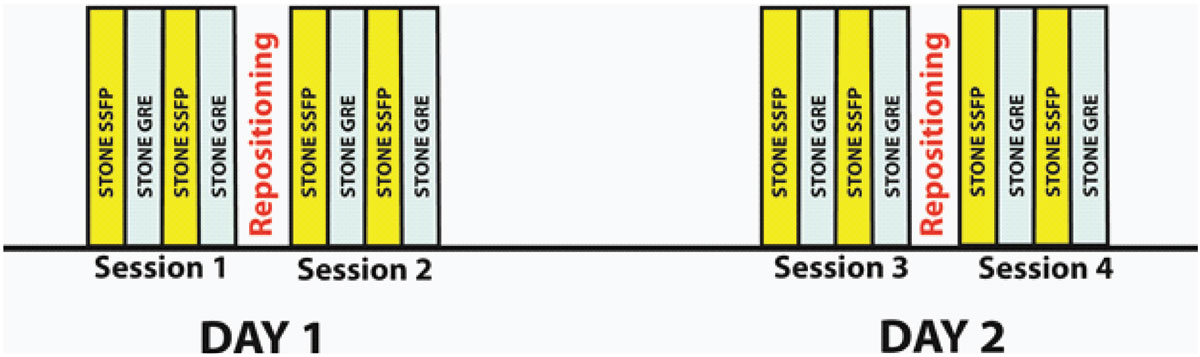
**Study protocol to assess within session, between session and between day (test/re-test) variability for native myocardial T1**.

Eleven healthy subjects (33 ± 16 years, 6 male) underwent non-contrast CMR imaging on 2 different days. Figure 1 shows the study design. Each subject was imaged twice with identical imaging protocol. After image prescription, the subjects were imaged using *STONE SSFP* with the following imaging parameters: In-plane resolution = 2.1 × 2.1 mm^2^, slice thickness = 8 mm, slice gap = 4 mm, Field of View = 320 × 320 mm^2^, TR/TE/α = 2.8 msec. / 1.38 msec. /70°, SENSE-rate = 2, linear ordering, 10 linear ramp-up pulses and acquisition window = 218.8 msec, bandwidth = 1879.7 Hz/pixel. *STONE GRE* sequence was acquired with following parameters: In-plane resolution = 2 × 2 mm2, slice thickness = 8 mm, slice gap = 4 mm, Field of View = 300 × 300 mm2, TR/TE/α = 3.9 msec. / 1.94 msec. /90°, SENSE-rate = 2.5, linear ordering, 10 linear ramp-up pulses and acquisition window = 166.6 msec., bandwidth= 1315.8 Hz/pixel. Imaging was repeated twice for each sequence. Subsequently, subjects were removed from the scanner and repositioned, followed by the same scan protocol. The same imaging protocol was repeated on a second day of scan. All imaging was performed in a 1.5T CMR scanner (Philips Achieva) using a 32-channel cardiac receiver coil array. T_1_ maps were created by voxel-wise fitting using a 2-parameter fit model after motion correction. The epicardial and endocardial contours in the left ventricle were manually drawn in 5 short axis-slices to calculate global and slice-based myocardial T_1_ values. Coefficient of variation analysis for each slice was generated to assess the variability within each session, between the sessions, between different days and for each subject.

## Results

**Figure 2:**
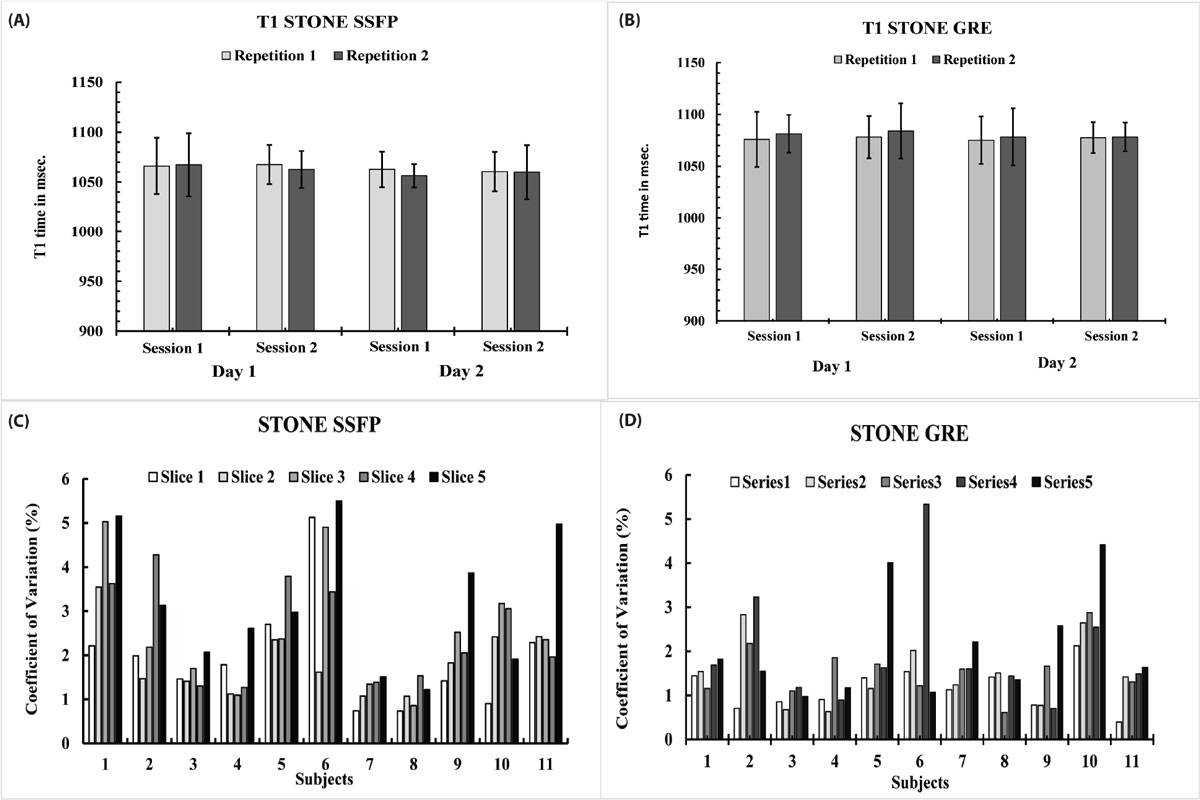
**Mean native T1 per repetition, session and day for STONE and coefficient of variation for different slices and subjects**.

Figure 2 shows mean T_1_ values for different imaging sessions, averaged over all subjects for STONE-SSFP and STONE-GRE. The CVs for all slices and *subjects* (figure 2) showed low variability for STONE-SSFP (2.4 ± 1.3%) and for STONE-GRE (1.65 ± 0.95%). The CVs for all slices and *days* showed a mean of 2.1 ± 1.45% for STONE SSFP and a mean of 1.5 ± 1.1% for Stone GRE. The CVs for all slices and *sessions* showed a mean of 1.7 ± 1.95% for STONE SSFP and a mean of 1.2 ± 1.3% for STONE GRE.

## Conclusions

Native myocardial T_1_ measurements by STONE-GRE and STONE-SSFP are very reproducible. These data suggest that STONE-SSFP and STONE-GRE should be considered for longitudinal studies to assess potential temporal changes in native T_1_ values for disease monitoring and/or during therapy.

